# Repeated Activation of Lung Invariant NKT Cells Results in Chronic Obstructive Pulmonary Disease-Like Symptoms

**DOI:** 10.1371/journal.pone.0147710

**Published:** 2016-01-26

**Authors:** Cheng-Chiu Tsao, Po-Nien Tsao, Yi-Guang Chen, Ya-Hui Chuang

**Affiliations:** 1 Department of Clinical Laboratory Sciences and Medical Biotechnology, College of Medicine, National Taiwan University, Taipei, Taiwan; 2 Department of Pediatrics, College of Medicine, National Taiwan University, Taipei, Taiwan; 3 The Research Center of Developmental Biology and Regenerative Medicine, National Taiwan University, Taipei, Taiwan; 4 Department of Pediatrics, Max McGee National Research Center for Juvenile Diabetes, Medical College of Wisconsin, Milwaukee, WI, 53226, United States of America; 5 Human and Molecular Genetics Center, Medical College of Wisconsin, Milwaukee, WI, 53226, United States of America; Institut Pasteur, FRANCE

## Abstract

Chronic obstructive pulmonary disease (COPD) is characterized by chronic airway inflammation, mucus hypersecretion, and emphysema, which lead to reduced lung function and breathlessness. The pathologies of COPD are due to an abnormal immune response. Invariant natural killer T (iNKT) cells are an important population of innate lymphocytes and have been implicated in the regulation of immune responses associated with a broad range of diseases including COPD. We have here analyzed the role of iNKT cells in a model of COPD induced by repeated intranasal administration of iNKT cell agonist α-galactosylceramide (α-GalCer). Our results demonstrated that mice that received repeated intranasal administration of α-GalCer had molecular and inflammatory features of COPD including airway inflammation with significant increases in infiltration of macrophages and lymphocytes, CD8^+^ T cells, as well as proinflammatory cytokines IL-6 and TNF-α. In particular, these mice also showed the presence of pulmonary emphysema, mucus production, and pulmonary fibrosis. Furthermore, neutralization of IL-4 reduced α-GalCer induced emphysema. This study indicates the importance of iNKT cells in the pathogenesis of COPD by an IL-4 dependent mechanism.

## Introduction

Global burden of human chronic lung diseases, such as asthma and chronic obstructive pulmonary disease (COPD), is increasing gradually. COPD is associated with cigarette smoking and exposure to various environmental pollutants. Viral and bacterial respiratory tract infections are also a risk factor for COPD [1–3]. COPD is characterized by a local inflammatory process manifested by activation of epithelial cells and resident macrophages and elevated levels of inflammatory cytokines such as IL-6, IL-8, and TNF-α [2, 4, 5]. It is associated with formation of mucous exudates within the lumens of small airways and lung parenchymal destruction leading to airspace enlargement [2, 4, 6]. COPD severity is associated with the accumulation of neutrophils, macrophages, natural killer (NK) cells, and T lymphocytes with a preponderance of the CD8^+^ subtype in the airways [7–9]. Emphysema, characterized by abnormal permanent enlargement of the air spaces, is the most important parameter to assess the presence and severity of COPD [1, 2, 10].

Invariant natural killer T (iNKT) cells are activated by glycolipid, such as α-galactosylceramide (α-GalCer), presented by CD1d. When activated, they produce large amounts of cytokines that can alter the strength and character of immune responses through crosstalk with dendritic cells, neutrophils, and lymphocytes, and by shifting cytokine responses to a T helper 1 (TH1), TH2 or TH17 phenotypes [11–13]. iNKT cells can also be activated by diverse microbial infections which have a profound impact on the development of inflammatory diseases. Microbial glycolipid, such as in *Sphingomonas* spp and *Borrelia burgdorferi*, directly activates iNKT cells [14, 15]. Some Toll like receptor (TLR) ligands, such as lipopolysaccharide and CpG, as well as mouse cytomegalovirus and herpes simplex virus 1 activate iNKT cells indirectly through myeloid antigen presenting cells [16–18].

Increased peripheral blood iNKT cells associated with an enhanced expression of the activating marker CD69 have been observed in patients with COPD compared to healthy subjects [19, 20]. In a mouse model of COPD induced by chronic cigarette smoke exposure, increased numbers of activated iNKT cells in the lung were also observed. Several features of COPD (e.g., inflammation and emphysema) were significantly suppressed in *Cd1d*^*-/-*^ and *Jα18*^*-/-*^ mice lacking iNKT cells [20], indicating a role of iNKT cells. In addition, mouse infected with Sendai virus, a mouse parainfluenza virus, develop long term airway inflammation associated with increased iNKT cells [19].

In this study, we investigated whether and how iNKT cell activation induces COPD-like symptoms. We repeatedly injected an iNKT cell agonist, α-GalCer, to activate lung iNKT cells and analyzed the features of the chronic airway inflammation in these mice. In addition, we studied the mechanism of how iNKT cell activation leads to emphysema. Our results demonstrate that iNKT cell activation induces COPD-like symptoms via IL-4 over-production.

## Materials and Methods

### Mice and α-GalCer administration

Female BALB/c mice, 6–8 weeks old, were obtained from the National Laboratory Animal Center and housed at the in-house animal care facility of the Animal Center of the College of Medicine, National Taiwan University under a 12 hour day-night-cycle and standardized environment. The protocol was approved by the Institutional Animal Care and Use Committee (IACUC) of National Taiwan University, College of Medicine and College of Public Health. Mice were intranasally administered with 2μg α-GalCer (0.2 mg/ml in 0.5% polysorbate 20 in PBS)(Funakoshi, Tokyo, Japan) once a week for 6 weeks. A vehicle control solution was prepared from a solution of 0.5% polysorbate 20 in PBS. Two weeks after the last α-GalCer administration, mice were sacrificed by pentobarbitol (50mg/kg) administration and then cervical dislocation and examined for pathological changes. For IL-4 neutralization, 150 μg of anti-IL-4 antibodies (clone 11B11, BioXcell, Lebanon, NH, USA) were intraperitoneally injected at 1 hour prior to every α-GalCer administration. BALB/c mice, but not C57BL/6 mice, were used in this study because the features of acute and chronic airway inflammation by α-GalCer administration were much higher in BALB/c mice than in C57BL/6 mice.

### Analysis of cellular composition and cytokines in the bronchoalveolar lavage fluid (BALF)

Cellular composition in the BALF was assessed as previously described [21]. Cytokines were evaluated by ELISA (Duoset) and chemokines were determined by Dot-blot-based mouse chemokine antibody array (Mouse Cytokine Array Kit) as recommended by the manufacturer (R&D Systems, Minneapolis, MN, USA). Lymphocyte subsets of BALF cells were determined by flow cytometry as previously described [22, 23]. Macrophages were isolated from BALF by adherence method.

### Flow cytometry

Cell population and cytokine secretion of iNKT cells were measured by flow cytometry. Before staining cells, with a previously defined optimal dilution of monoclonal antibodies (Abs), the cells were pre-incubated with anti-CD16/32 (clone 93) to block non-specific FcRγ binding. The following Abs were used in this study: anti-CD3, anti-CD4, anti-CD8, and anti-CD49b (Biolegend, San Diego, CA, USA). For intracellular staining, BALF cells were incubated with brefeldin A (10 μg/ml) (BD Biosciences, San Diego, CA, USA) at 37°C for 1 hour then incubated with anti-CD16/32 Abs, followed by staining with PerCP/Cy5.5-conjugated CD3 and PE-conjugated PBS57 loaded CD1d tetramer (originally produced by the NIH tetramer facility, and supplied through Dr. David Serreze), permeabilized with Cytofix/Cytoperm reagent (BD Biosciences), and stained with Alexa Fluor® 488-conjugated anti-IFN-γ (clone XMG1.2), Alexa Fluor® 488-conjugated anti-IL-4 (clone 11B11), or rat IgG1 isotype control (clone R3-34) (BD Biosciences). Stained cells were assessed on a FACSCalibur (BD Biosciences) using FlowJo softwares (Tree Star, Inc., Ashland, OR, USA).

### Measurements of pulmonary function

Pulmonary function was measured by measuring the changes of airway resistance, dynamic compliance, and airway elastance in response to increasing doses of aerosolized methacholine (3.125–12.5 mg/ml) (Sigma-Aldrich) using a Buxco Pulmonary Mechanics System (Buxco Electronics, Wilmington, NC). Mice were anesthetized, tracheostomized, and mechanically ventilated at a rate of 150 breaths/min, a tidal volume of 0.3ml, and a positive end-expiratory pressure of 3–4 cmH_2_O with a computer-controlled small animal ventilator. Pulmonary parameters were recorded for 3 minutes after 3-minute exposure of aerosol methacholine and analyzed by BioSystem XA software.

### Tissue preparation

Lungs were perfused with PBS through the right ventricle to remove blood from the vascular bed. Ten percent buffered formalin solution was instilled through the tracheal cannula at a constant pressure of 20 cmH_2_O to inflate and fix the lung. Specimens were immersed in 10% buffered formalin solution overnight and dehydrated in a graded series of ethanol solutions. Tissue was embedded in paraffin, and sections were cut at 5-μm thickness for hematoxylin and eosin (H&E) staining, periodic acid-Schiff (PAS) staining and Massion’s trichrome staining for the examination of pulmonary morphometry, mucus production, and fibrosis.

### Morphometric analysis

For mean linear intercept (MLI) analysis, digital images of airways were acquired with a microscope and imported into MetaMorph® Imaging System software version 7.7 (Universal Imaging Corp., Downingtown, PA, USA). Briefly, a series of grid lines was laid over each photomicrograph to determine the number of times those lines were intercepted by alveolar tissue, with L_m_ = L/L_i_, where L was the total length of the lines in the grid field and L_i_ was the total number of times those lines were intercepted.

### MMP12 detection in cytokine stimulated bone marrow–derived macrophages (BM-Mϕ)

Bone marrow cells from femurs and tibias of BALB/c mice were incubated with 20% L929 conditioned medium for 7 days to allow differentiation and maturation of macrophages. The purity of macrophages stained with anti-Ly6G and anti-F4/80 antibodies and determined by flow cytometry (Ly6G^-^F4/80^+^) and cell morphology was ≥ 99%. BM-Mϕ were stimulated with different concentrations of IL-4 and IFN-γ for 18 hours. Cells were collected for RNA extraction and expression of *Mmp12* was analyzed by qRT-PCR.

### Quantitative real time RT-PCR analysis

Total RNA was isolated from liver tissue using Tri^zol^ reagent (Invitrogen Life Technologies). Total RNA was reverse transcribed to cDNA with random primers using the Reverse Transcription System kit following manufacturer’s instructions (Thermo Scientific Fermentas, Rockford, IL, USA). PCR was performed on a 7500 Fast Real-Time PCR System (Applied Biosystems). SYBR Green DNA-binding dye was used in the amplification reactions. Fluorescence signals were analyzed during each cycle (pre-treatment 2 minute at 50°C, initial denaturation 10 minute at 95°C and denaturation 15 seconds at 95°C and annealing/extension 1 minute at 60°C for 40 cycles). Data were normalized to internal β-actin expression. The primers used in the qPCR assay are listed in Table 1.

**Table 1:** Primers used in this study.

Oligo Name		Sequence
β-actin	Forward	5'-CACAGTGTTGTCTGGTGGTA -3'
	Reverse	5'-GACTCATCGTACTCCTGCTT -3'
Muc5ac	Forward	5'-AGAATATCTTTCAGGACCCCTGCT -3'
	Reverse	5'-ACACCAGTGCTGAGCATACTTTT -3'
collagen III	Forward	5'- GTTCTAGAGGATGGCTGTACTAAACACA -3'
	Reverse	5'-TTGCCTTGCGTGTTTGATATTC -3'
IL-6	Forward	5'-CTCTGGGAAATCGTGGAAATG -3'
	Reverse	5'-AAGTGCATCATCGTTGTTCATACA -3'
TNF-α	Forward	5'-CCCCAAAGGGATGAGAAGTTC -3'
	Reverse	5'-TGAGGGTCTGGGCCATAGAA -3'
MMP-12	Forward	5’-GAAACCCCCATCCTTGACAA-3’
	Reverse	5’-TTCCACCAGAAGAACCAGTCTTTAA-3’

### Statistical analysis

Data are presented as mean ± SEM. Statistical analysis was performed with GraphPad Prism (GraphPad Software). Data were analyzed by Kruskal-Wallis one-way ANOVA followed Dunn's post test and by Mann-Whitney U test. *, p<0.05; **, p<0.01; ***, p<0.001 were considered to be statistically significant.

## Results

### Intranasal α-GalCer administration activates iNKT cells and induces acute airway inflammation

**Fig 1:**
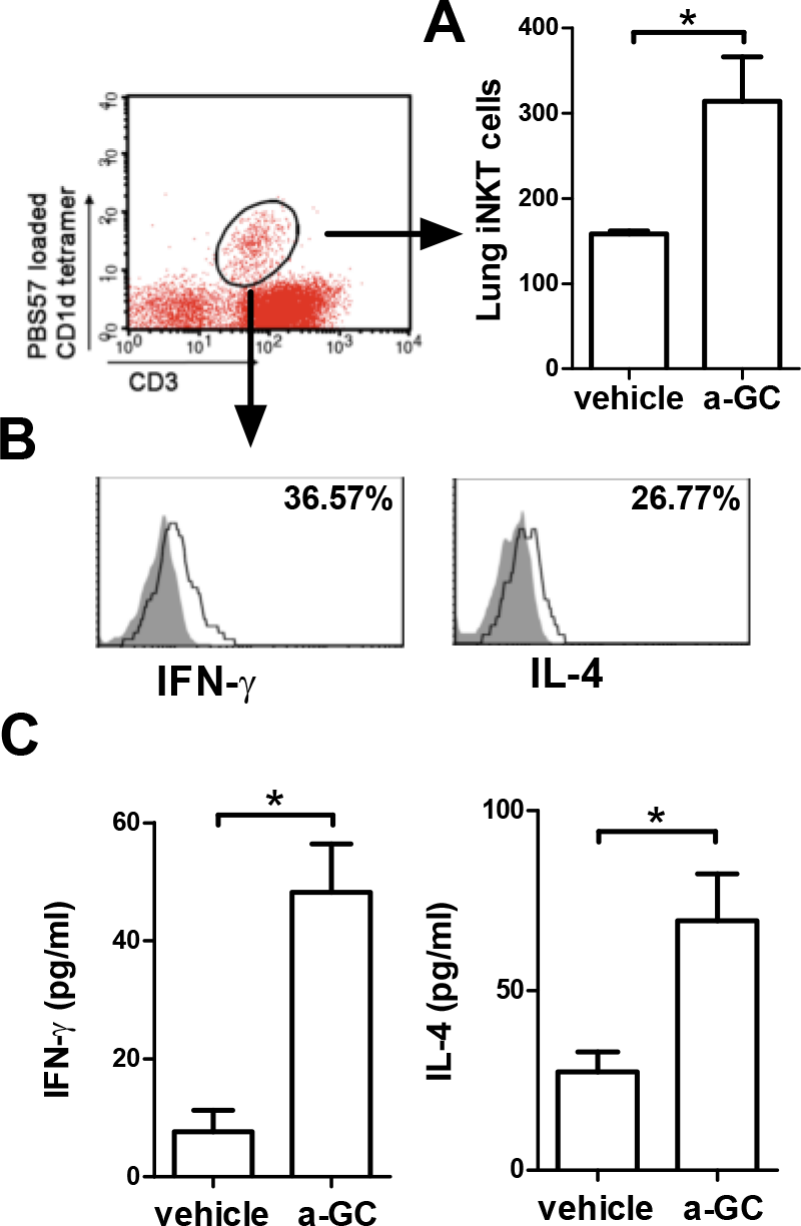
Intranasal α-GalCer administration activates lung iNKT cells to secrete cytokines. Mice were intranasally administered with vehicle or α-GalCer. Two hours later, BALF was collected. (A) The absolute numbers of iNKT (CD3^+^PBS57 loaded CD1d tetramer^+^) cells were measured. (B) IFN-γ and IL-4 expression from iNKT cells was assayed by intracellular staining with anti-mouse IFN-γ and IL-4 Abs. The shaded area is an isotype control; the open area reflects cytokine expression. The percentage of IFN-γ and IL-4-producing iNKT cells were shown. (C) IL-4 and IFN-γ levels in the BALF were measured by ELISA. a-GC, α-GalCer. n = 5 mice for each group. *, p<0.05 using Mann-Whitney U test.

In our previous study, we found intranasal administration of α-GalCer, the most studied glycolipid that activates iNKT cells, induced cytokine production and airway inflammation within hours in wild-type mice but not in iNKT cell deficient CD1d knockout mice [21]. To get a better understanding of iNKT cell mediated airway inflammation, we carried out detailed analyses of immunological changes induced by a single intranasal α-GalCer administration. Two hours after α-GalCer administration, the numbers of BALF iNKT (CD3^+^CD1d tetramer ^+^) cells were increased (Fig 1A) and they produced IFN-γ and IL-4 (Fig 1B). In addition, IFN-γ and IL-4 in the bronchoalveolar lavage fluid (BALF) were increased in mice administered with α-GalCer (Fig 1C).

**Fig 2:**
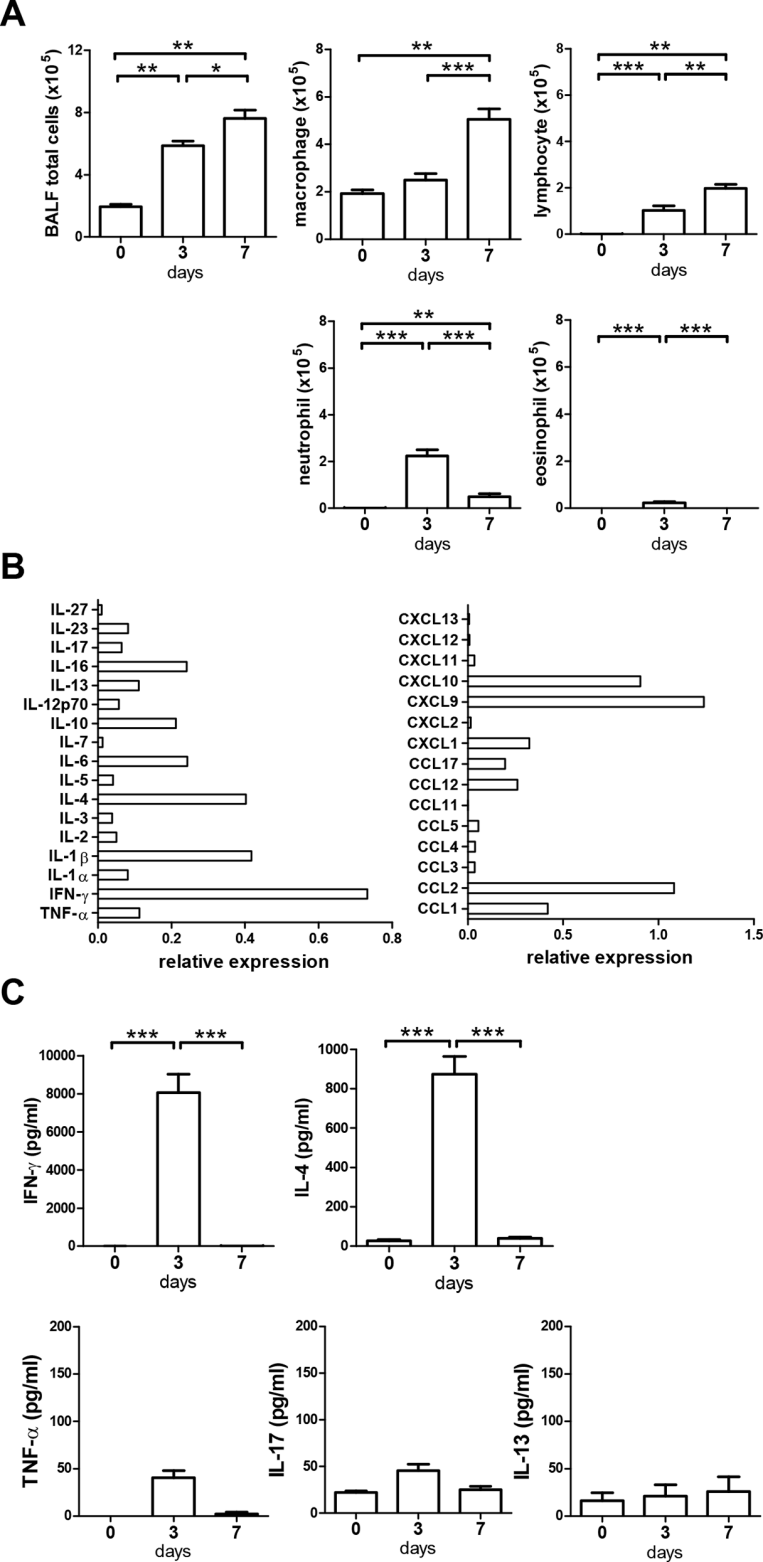
Intranasal α-GalCer administration induced acute inflammation in the lung. Mice were intranasally administered with vehicle or α-GalCer. BALF was collected at days 0, 3 and 7. (A) BALF total cells and the absolute numbers of macrophages, lymphocytes, neutrophils, and eosinophils were measured at the indicated time points. (B) The relative expressions of cytokines and chemokines compared to positive control (set to a value of 1) of the assay kit in BALF at 3 days after α-GalCer administration of mice were determined by a dot blot immunoassay. Samples were pooled from 5–8 mice in two independent experiments. (C) Cytokines IFN-γ, IL-4, TNF-α, IL-17, and IL-13 in the BALF were assayed by ELISA. n = 5–8 mice for each group. *, p<0.05; **, p<0.01; ***, p<0.001 using Kruskal-Wallis one-way ANOVA followed Dunn's post test.

We then analyzed the airway inflammation at 3 and 7 days after α-GalCer administration. BALF total cells were increased. Among them, macrophages and lymphocytes were gradually increasing after α-GalCer administration; nerutophils were increased at 3 days but trended down at 7 days; eosinophils were slightly increased at 3 days (Fig 2A). With cytokine and chemokine array assay, we found enhanced expression of several cytokines and chemokines in the BALF at 3 days after α-GalCer administration while undetectable at day 0 (Fig 2B and data not shown). IFN-γ and IL-4 were highly expressed at 3 days and back to baseline at 7 days after α-GalCer administration. TNF-α and IL-17 were slightly increased at 3 days after α-GalCer administration. IL-13 was not increased (Fig 2C). Taken together, these results combined with our previous data suggested that α-GalCer administration activated iNKT cells to secrete cytokines such as IL-4 and IFN-γ which then recruited inflammatory cells including neutrophils, lymphocytes and macrophages to the lung in wild type mice.

### Decreased lung function and increased airway inflammation in mice repeated intranasally administered with α-GalCer

**Fig 3:**
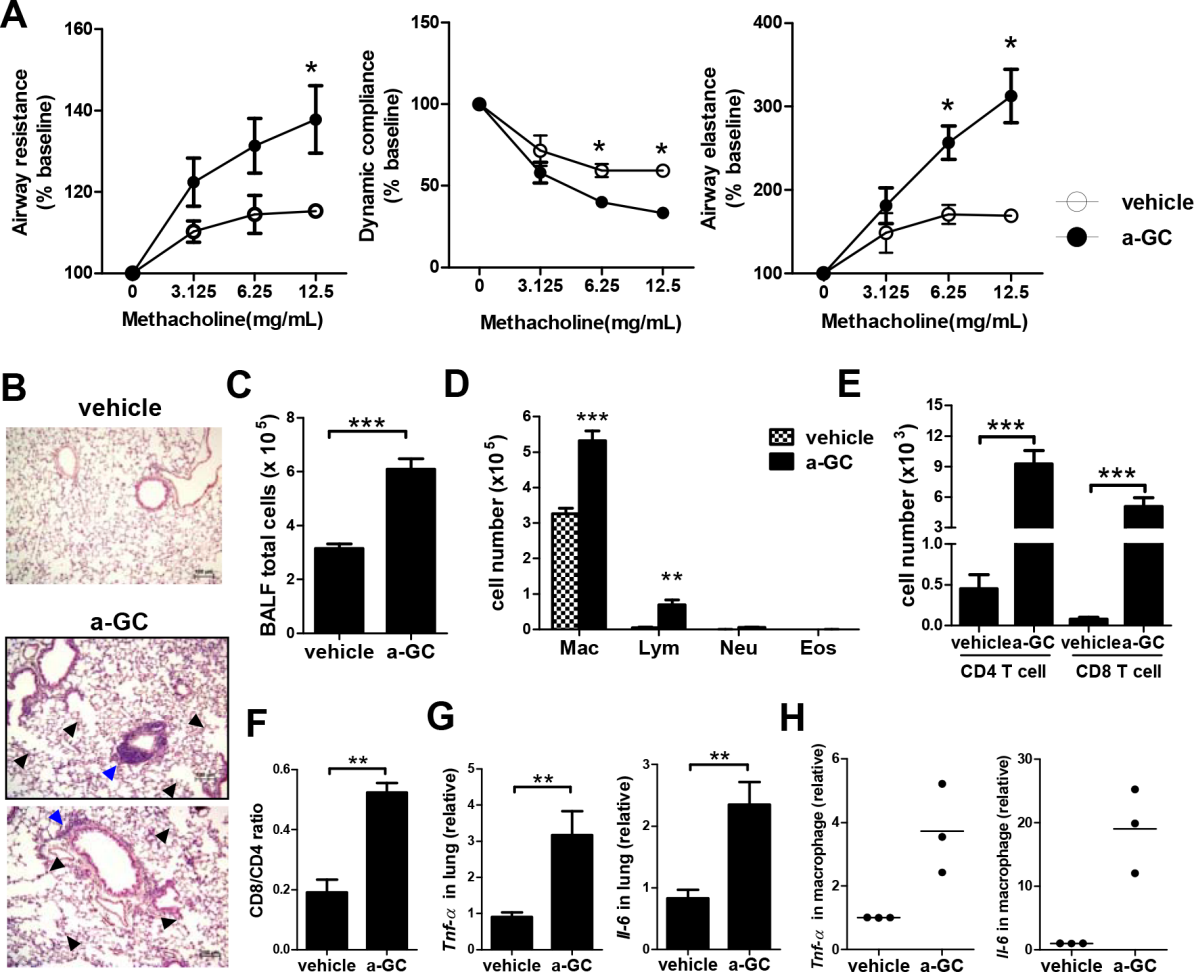
Increased airway inflammation in mice repeatedly administered with α-GalCer. Mice received intranasal α-GalCer administration once a week for 6 weeks and airway inflammation was measured 2 weeks after the last administration. (A) Analysis of lung function. Changes in airway resistance, dynamic compliance, and airway elastance were measured on mechanically ventilated mice, in response to increasing doses of methacholine. (B) Representative results of H-E examination of lung sections (x100 magnification). Blue arrowheads indicate cell infiltration in the lung. Black arrowheads indicate airspace enlargement in the lung. (C) The total numbers of cells present in BALF. (D) The absolute numbers of macrophages, lymphocytes, neutrophils, and eosinophils, present in BALF. Mac, macrophage; Lym, lymphocyte; Neu, neutrophil; Eos, eosinophil. (E) The absolute numbers of CD4^+^ and CD8^+^ T cells were examined. (F) CD8/CD4 ratios of T cells were measured. (G) Lung tissues were isolated and the expression of TNF-α and IL-6 was detected by quantitative RT-PCR. n = 9–14 mice for each group. (H) Macrophages from BALF were isolated and their expression of TNF-α and IL-6 was detected by quantitative RT-PCR. Individual symbols represent a sample pooled from 3–5 mice and the horizontal lines represent the mean values. a-GC, α-GalCer. *, p<0.05; **, p<0.01; ***, p<0.001 using Mann-Whitney U test.

To understand the role of lung iNKT cells in the pathogenesis of chronic lung disease, mice were intranasally administered with α-GalCer once a week for 6 weeks and features of chronic airway inflammation were examined at 2 weeks after the last administration. Mice repeated intranasally administered with α-GalCer did not have abnormal appearance and behavior (data not shown). As shown in Fig 3A, a decline of lung function including increased airway resistance, reduced dynamic complicance, and increased airway elastance was observed in mice repeatedly administered with α-GalCer. Histological examination of H&E-stained lung tissue sections of mice repeatedly administered with α-GalCer demonstrated apparent cell infiltration in the lung. Aggregates appeared to be located around the airways. These cell infiltrates were not found in lungs of vehicle-treated controls (Fig 3B). BALF total cells were significantly increased in mice repeatedly administered with α-GalCer (Fig 3C). Among them, macrophages and lymphocytes were significantly increased (Fig 3D). Both CD4^+^ and CD8^+^ T cells were increased in α-GalCer administered mice (Fig 3E) with an increased CD8/CD4 ratio (Fig 3F), suggesting a preponderance of the CD8^+^ subtype in T cells. Proinflammatory cytokines, TNF-α and IL-6, in the lung of α-GalCer administered mice were increased compared to those of vehicle administered mice (Fig 3G). In addition, increased expression of TNF-α and IL-6 was also noted in macrophages from the BALF of α-GalCer administered mice (Fig 3H).

### Pulmonary emphysema, mucus production, and fibrosis in mice receiving repeated intranasal administration of α-GalCer

**Fig 4:**
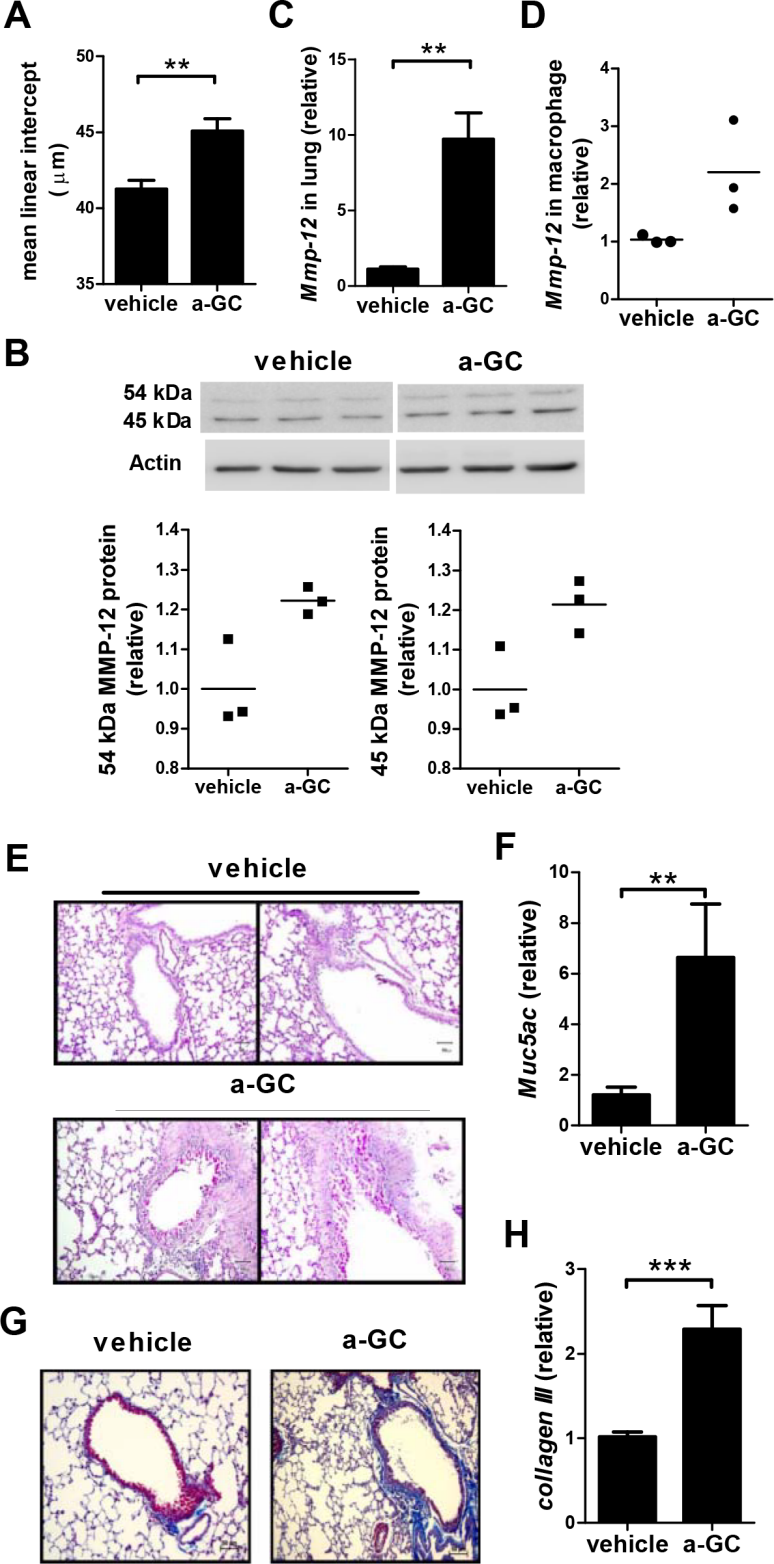
The presence of emphysema, mucus production and fibrosis in mice with repeated intranasal administration of α-GalCer. Mice were intranasally administered with α-GalCer once a week for 6 weeks and histopathological changes were examined 2 weeks after the last administration. (A) The mean linear intercept of the alveolar septa was measured. (B) The upper panels show the western blot results of 54 kDa and 45 kDa MMP12 protein expression in the lung and summarized in the lower panels. Actin was used as an internal control. Each symbol represents an individual mouse and the horizontal lines represent the means. (C) The expression of *Mmp-12* in lung tissues was analyzed by quantitative RT-PCR. (D) The expression of *Mmp-12* in lung macrophages was measured by quantitative RT-PCR. Each symbol represents cells pooled from 2–3 mice and the horizontal lines represent the means of three symbols. (E) Representative sections of the lung stained with PAS for analysis of mucus-containing cells. (200x magnification). (F) The expression of *Muc5ac* in lung tissues was analyzed by quantitative RT-PCR. (G) Representative results of Massion’s trichrome staining of lung sections (200x magnification, blue color). (H) The expression of *collagen III* in lung tissues was evaluated by quantitative RT-PCR. n = 9–14 mice for each group. a-GC, α-GalCer. **, p<0.01; ***, p<0.001 using Mann-Whitney U test.

Emphysema is a pulmonary disease characterized by abnormal permanent enlargement of the air spaces accompanied by destruction of the alveolar walls, which is the most important parameter to assess the presence and severity of COPD [1, 2, 10]. The mean linear intercept (Lm) is a measure of the surface area to volume ratio and is the most commonly reported metric of emphysema [24]. As shown in Fig 3A, the enlargement of the air space was clearly observed in α-GalCer administered mice compared to vehicle-treated control. In addition, significantly increased mean linear intercept (Lm) in the lung tissue of α-GalCer administered mice was noted compared to the vehicle-treated mice (45.07 μm±2.57 vs 41.27 μm±1.77, p<0.01) (Fig 4A). Matrix metalloproteinases (MMP)-12 is an important proteinase in the development of emphysema [25]. It is secreted as a 54 kDa inactive pro-enzyme, which is activated by proteolytic cleavage of the prodomain followed by processing into an active enzyme of 45 kDa and then a 22 kDa [26]. MMP12 protein and mRNA were also significantly increased in lung tissues of α-GalCer administered mice (Fig 4B and 4C). In addition, increased expression of MMP12 was also noted in macrophages from the BALF of α-GalCer administered mice (Fig 4D). We then examined mucus production of the lung tissue. Positive PAS staining mucus producing cells were found only in α-GalCer administered mice but not in the vehicle-treated control (Fig 4E). Furthermore, the expression of *Muc5ac* in the lung of the α-GalCer administered mice was significantly higher than that in vehicle treated mice (Fig 4F). α-GalCer administered mice also exhibited mild lung fibrosis as highlighted by Massion’s trichrome staining (Fig 4G). Of note, collagen III was significantly increased in α-GalCer administered mice (Fig 4H).

### IL-4 enhanced MMP12 production in macrophages

**Fig 5:**
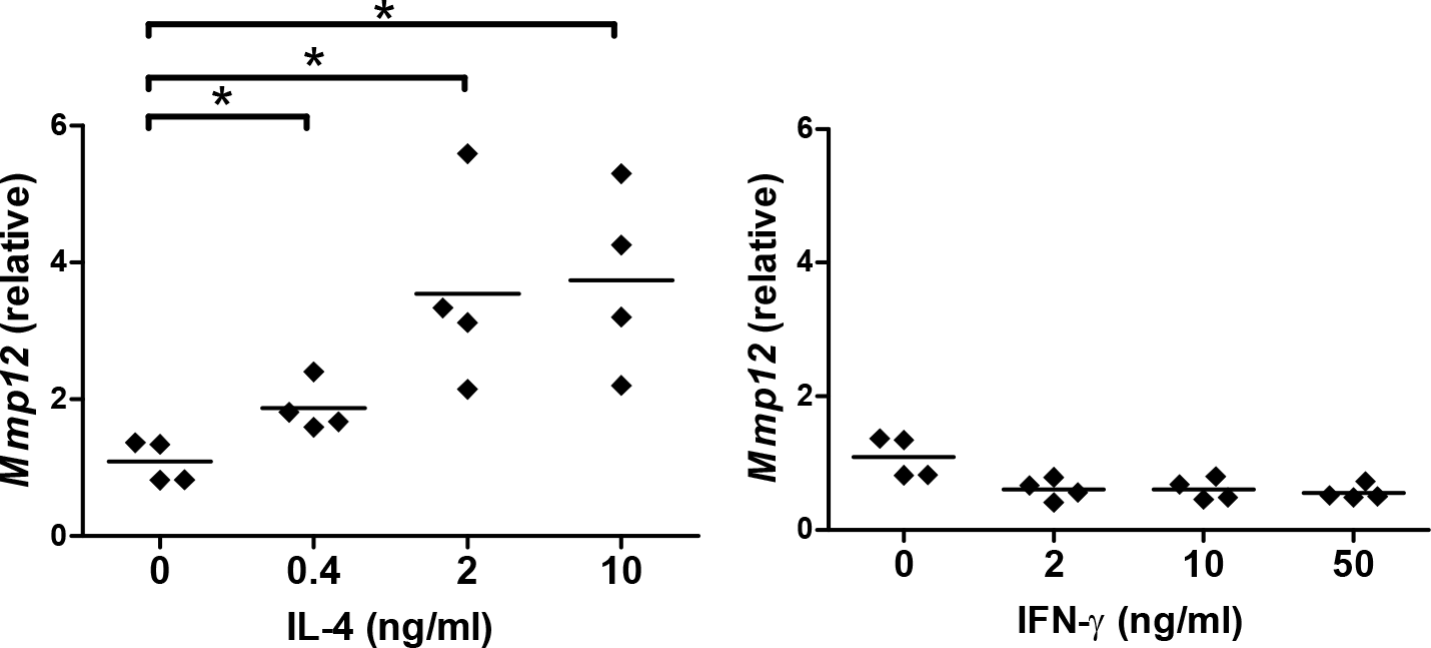
IL-4 enhances *Mmp12* expression in macrophages. Bone marrow derived macrophages were stimulated with different concentrations of IL-4 or IFN-γ and the expression of *Mmp12* was measured by quantitative RT-PCR. Summarized results of 4 independent experiments are shown. Each symbol represents an individual experiment and the horizontal lines represent the means. *, p<0.05 using Kruskal-Wallis one-way ANOVA followed Dunn's post test.

We then studied the mechanism of how repeated α-GalCer administration led to emphysema. We hypothesized that the IFN-γ and/or IL-4 production by activated iNKT cells enhanced MMP12 expression and the development of emphysema. As MMP-12 is mainly expressed by macrophages [27], we first investigated whether IFN-γ and/or IL-4 affected MMP12 expression in macrophages. This was done by stimulating bone marrow derived macrophages with IL-4 or IFN-γ and measuring their *Mmp12* expression. The results showed that IL-4 was a potent inducer of *Mmp12* mRNA expression, whereas IFN-γ slightly downregulated *Mmp12* mRNA expression in bone marrow derived macrophages (Fig 5).

### IL-4 neutralization reduced α-GalCer induced emphysema

**Fig 6:**
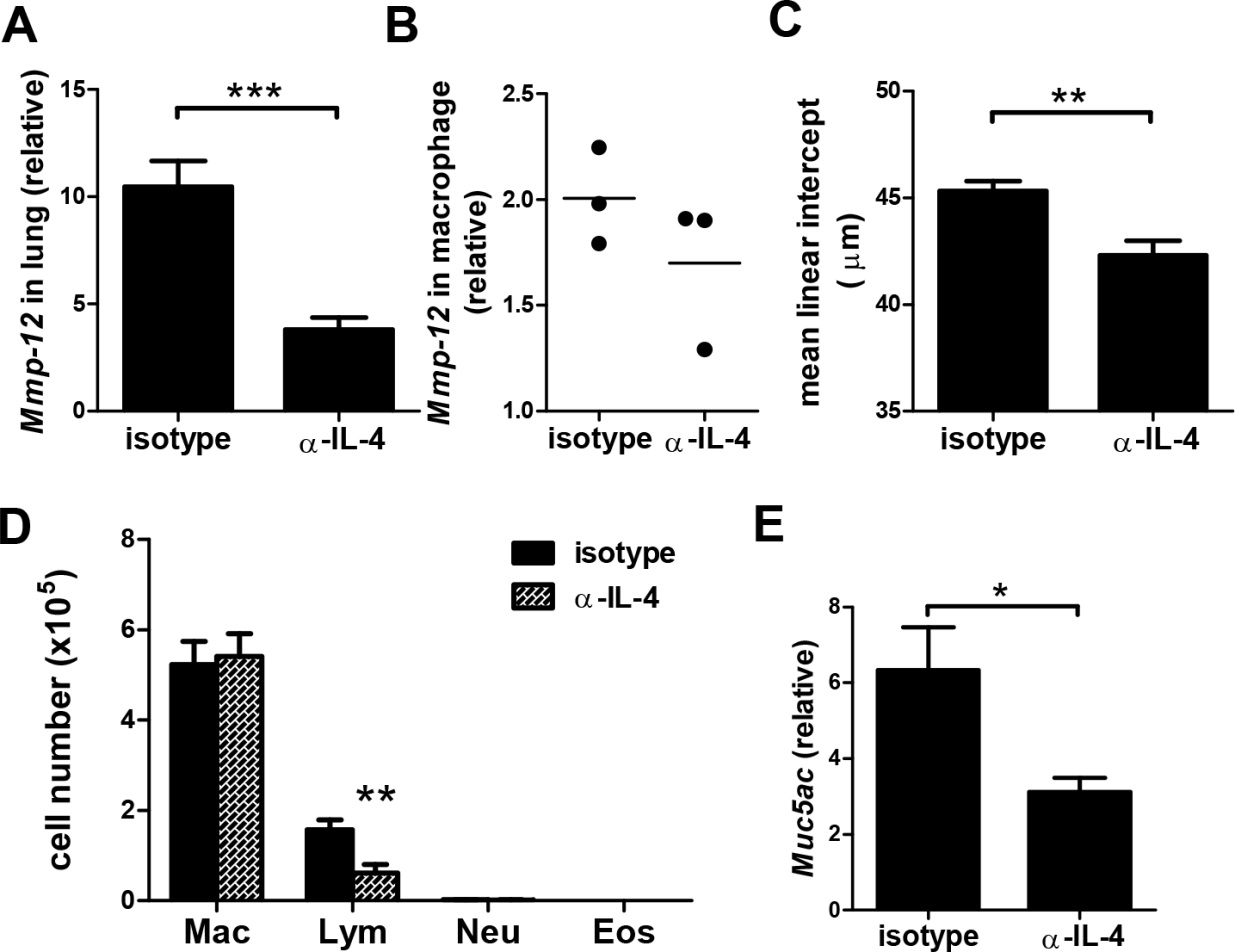
Anti-IL-4 Abs reduced emphysema and mucus production in mice repeatedly administered with α-GalCer. Mice were injected with neutralizing Abs to IL-4 or isotype controls 1 hour prior to each α-GalCer administration. Histopathological examination was measured 2 weeks after the last α-GalCer administration. (A) The expression of *Mmp-12* in lung tissues was analyzed by quantitative RT-PCR. (B) The expression of *Mmp-12* in lung macrophages was analyzed by quantitative RT-PCR. (C) The mean linear intercept of the alveolar septa was measured. (D) The absolute numbers of macrophages, neutrophils, eosinophils, and lymphocytes were measured. Mac, macrophage; Lym, lymphocyte; Neu, neutrophil; Eos, eosinophil. (E) The expression of *Muc5ac* in lung tissues was detected by quantitative RT-PCR. n = 9–16 mice for each group. *, p<0.05; **, p<0.01; ***, p<0.001 using Mann-Whitney U test.

We then studied whether IL-4 in the α-GalCer induced COPD-like symptom model increased MMP12 expression and enlarged airway space. We administered mice with neutralizing antibodies to IL-4 one hour prior to every α-GalCer administration. Two weeks after the last administration, mice were analyzed for features of COPD. As shown in Fig 6A, *Mmp12* expression in lung was significantly reduced in mice treated with anti-IL-4 Abs. *Mmp12* expression in macrophages was also reduced in mice treated with anti-IL-4 Abs (Fig 6B). In addition, the mean linear intercept was significantly reduced in anti-IL-4 Abs treated α-GalCer administered mice than in isotype Abs-treated mice (Fig 6C). Lymphocytes in the BALF were significantly decreased in α-GalCer administered mice that were also treated with anti-IL-4 Abs while the numbers of macrophages were not changed (Fig 6D). Furthermore, the expression of *Muc5ac* in the lung of the anti-IL-4 Abs treated mice was lower than isotype Abs treated mice (Fig 6E). These results suggested that IL-4 contributed to the pathogenesis of iNKT cell induced COPD-like symptoms.

## Discussion

In this study, we showed that repeated intranasal α-GalCer administration induced airway inflammation in mice. Total inflammatory cells in the BALF were increased in mice repeatedly administered with α-GalCer. Among them, macrophages and lymphocytes were significantly increased. Importantly, increased CD8^+^ T cells were observed in repeatedly α-GalCer administered mice. In addition, increased proinflammatory cytokines, mucus production, airspace enlargement, and pulmonary fibrosis were also noted in mice with repeated intranasal α-GalCer administration. All of these features in repeatedly α-GalCer administered mice were similar to pathological hallmarks of COPD. Furthermore, neutralization of IL-4 reduced α-GalCer induced emphysema. Collectively, these data suggested that chronic activation of iNKT cells in the airways lead to IL-4 mediated COPD-like symptoms.

In human COPD, increased numbers of neutrophils, macrophages and lymphocytes and elevated levels of numerous inflammatory cytokines such as IL-6, IL-8, and TNF-α are noted in the lungs of patients [2, 4, 5, 7]. It is suggested that release of proinflammatory cytokines and chemokines by airway epithelial cells and alveolar macrophages elicits the recruitment of neutrophils, macrophages and lymphocytes to the lungs [2, 4, 5]. Activated neutrophils and macrophages cause lung destruction through the release of oxygen radicals and proteolytic enzymes such as matrix metalloproteinases (MMPs), including MMP-12 [28]. Mice lacking MMP-12 are completely protected against emphysema [25].

Previously, we and others showed that α-GalCer administration before OVA immunization increases serum levels of IgE, Th2 cytokines, airway hyperreactivity (AHR), and airway eosinophilia after OVA rechallenging in the OVA-alum immunized murine model of allergic asthma [21, 29–31]. In patients with COPD, the frequency of activated iNKT cells is increased compared to controls [19, 20]. In mice chronically exposed to cigarette smoke, activated iNKT cells accumulate in the lungs and significantly contribute to the pathogenesis of COPD [20]. Importantly, Kim et al. show that viral infection induces chronic airway inflammation which is driven by macrophages and requires the presence of iNKT cells, suggesting that iNKT cells activated by virus infection induce alternative activation of macrophages, which then drive chronic lung disease [19]. These results combined with ours suggest that iNKT cell activation is critical in the development of chronic lung diseases including asthma and COPD.

In this study, we showed that mice repeatedly administered with α-GalCer produced high levels of IL-4 and features of COPD. Administration of anti-IL-4 Abs was found to reduce MMP12 expression and emphysema in α-GalCer administered mice. These results suggest a critical role of IL-4 in the pathogenesis of iNKT cell activation induced COPD. Upregulation of IL-4 has been documented in emphysematous human lung tissues [32] and cigarette smoke-exposed murine lungs [33]. Although the studies of IL-4 in COPD are limited, our results show that IL-4 can enhance the production of MMP12 and lung destruction.

In this study, *Mmp12* mRNA expression in α-GalCer administered mice had a 10-fold increase in lung tissue but it is only increased by 2-fold in macrophage. MMP-12 is dominantly produced by macrophages; however, other cells such as human airway smooth muscle cells also express and secrete MMP-12 [34]. Although *Mmp12* mRNA expression in the lung tissue of the α-GalCer administered mice was upregulated by 10-fold, the protein levels of the 54 kDa latent form and the 45 kDa active form were only increased by 1.2-fold. These results were similar to previous studies on MMP-12 release by macrophages and smooth muscle cells [27, 34]. The difference in levels of mRNA and protein suggests a complex regulation of MMP-12 translation and secretion. Otherwise, it could be due to the timing of sample collection.

To further study the mechanisms of human COPD, animal models have to be established. Cigarette smoke exposure to mice is the most frequently used animal model of COPD. However, the induction of either emphysema or small airway remodeling in cigarette smoke induced COPD model requires more than 6 months of smoke exposure [35]. In this study, we showed that it requires only 6 weeks to induce a COPD like airway inflammation in mice by repeated administration with α-GalCer, and thus provide an alternative approach to study this disease.

In conclusion, repeated activation of iNKT cells by α-GalCer administration in the airways can lead to chronic lung disease similar to human COPD. This study demonstrates a role of iNKT cell activation in chronic airway inflammatory disease and repeated airway α-GalCer administration can be used as a new model for the study of COPD.

## Abbreviations

α-GalCer: α-galactosylceramide
BALF: bronchoalveolar lavage fluid
COPD: chronic obstructive pulmonary disease
iNKT: Invariant natural killer T
MMP: matrix metalloproteinases
